# Neutrophil Extracellular Traps Go Viral

**DOI:** 10.3389/fimmu.2016.00366

**Published:** 2016-09-19

**Authors:** Günther Schönrich, Martin J. Raftery

**Affiliations:** ^1^Institute of Medical Virology, Helmut-Ruska-Haus, Charité − Universitätsmedizin Berlin, Berlin, Germany

**Keywords:** neutrophil extracellular traps, immunopathogenesis, neutrophils, viruses, viral immune evasion

## Abstract

Neutrophils are the most numerous immune cells. Their importance as the first line of defense against bacterial and fungal pathogens is well described. In contrast, the role of neutrophils in controlling viral infections is less clear. Bacterial and fungal pathogens can stimulate neutrophils extracellular traps (NETs) in a process called NETosis. Although NETosis has previously been described as a special form of programmed cell death, there are forms of NET production that do not end with the demise of neutrophils. As an end result of NETosis, genomic DNA complexed with microbicidal proteins is expelled from neutrophils. These structures can kill pathogens or at least prevent their local spread within host tissue. On the other hand, disproportionate NET formation can cause local or systemic damage. Only recently, it was recognized that viruses can also induce NETosis. In this review, we discuss the mechanisms by which NETs are produced in the context of viral infection and how this may contribute to both antiviral immunity and immunopathology. Finally, we shed light on viral immune evasion mechanisms targeting NETs.

## Introduction

As the first line of defense against invading pathogens, neutrophils have a broad arsenal of antimicrobial functions (1). For example, activated neutrophils release granules containing antimicrobial molecules and produce reactive oxygen species (ROS) by oxidative burst. An alternative antimicrobial function of neutrophils is based on a special type of programmed cell death called NETosis that is distinct from apoptosis and necrosis (2, 3). During NETosis, the nuclei of neutrophils lose their characteristic shape, and chromatin decondensation takes place (4). Subsequently, the membranes of the nucleus and the granules disintegrate, allowing the mixing of their content. Finally, neutrophils release neutrophil extracellular traps (NETs). NETs are net-like structures that are composed of chromatin and endowed with granule proteins. They bind to, entrap, and often kill certain pathogens. NETs are released particularly in response to large microbial structures that cannot be easily phagocytosed such as *Candida albicans* hyphae and *Mycobacterium bovis* aggregates (5).

Classical NETosis requires the generation of ROS by NADPH oxidase. However, mitochondrial ROS production in the absence of a functional NADPH oxidase is sufficient to trigger NETosis (6). Moreover, a very rapid and ROS-independent form of NETosis is triggered by *Staphylococcus aureus* (7). Thus, depending on the stimulus NADPH is not always required for NET formation (8). Similar to necrosis and apoptosis, there are different forms of NETosis (9, 10). For example, it has been observed that NET formation can occur without concomitant neutrophil death (7, 11–14). The physiological and pathological meanings of these different NETosis forms still have to be elucidated.

Only recently, it was recognized that NETs are also generated during viral infection (15–17). Evidence is accumulating that neutrophils play a role in antiviral immune responses (18). These virus-induced NETs can both control the virus and damage the host (19). In this review, we focus our attention on the physiological and pathological relevance of virus-induced NETosis.

## Viral NET Induction

**Figure 1:**
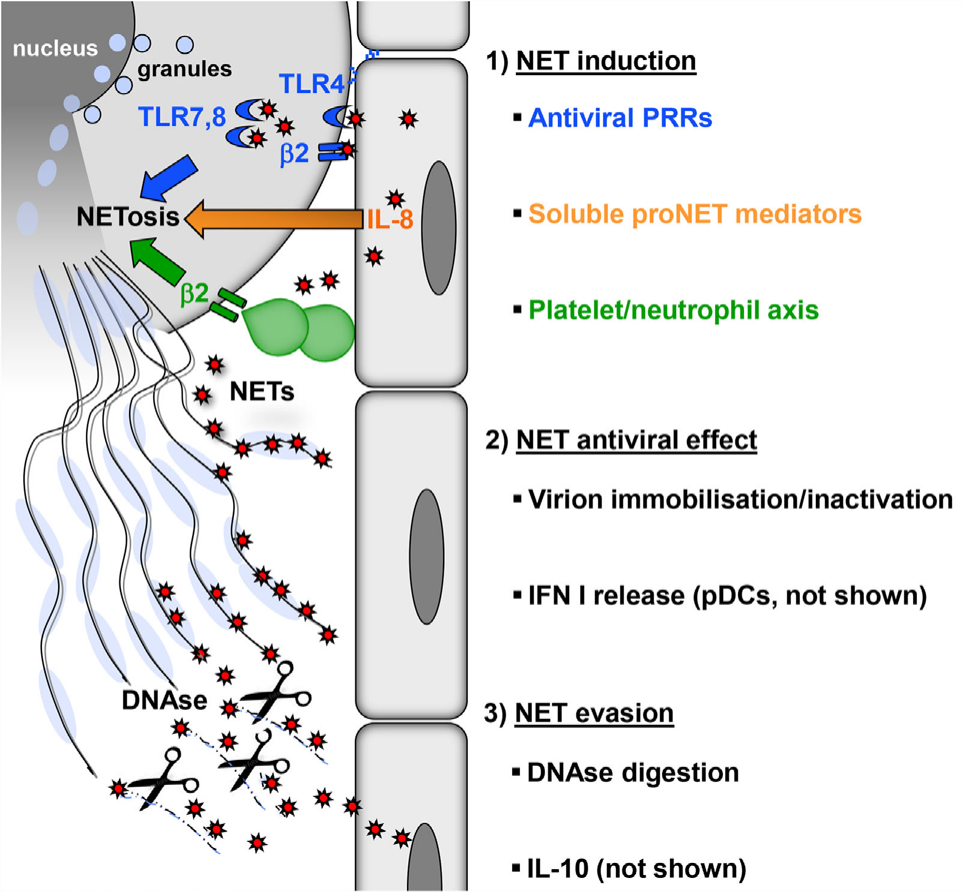
**Induction, antiviral effect, and viral evasion of NETs**. (1) Formation of NETs is induced directly by virions (red) through PRRs (blue) expressed by neutrophils on the surface (TLR4, β2 integrins) or in endosomes (TLR7, 8) or indirectly by proinflammatory mediators (e.g., IL-8), which are released from virus-infected cells (orange). In addition, viral activation of the platelet/neutrophil axis can trigger NETosis (green). As a consequence, granules fuse with the nucleus, which subsequently loses its characteristic lobulated shape and ruptures. Finally, neutrophils rupture releasing sticky strings of NETs. (2) NETs have antiviral effects by immobilizing or inactivating free virions, thereby preventing viral spread. NETs also potentiate the release of type I interferon by pDC (not shown), thus increasing the resistance of local cells to further infection. (3) Digestion of the DNA backbone by DNases releases trapped virions. These virions, if not already inactivated, opsonized, or degraded, can attempt to infect further cells. Moreover, viruses can interfere with NETosis by inducing cellular IL-10 or by expressing viral IL-10 homologs (not shown).

Many viruses stimulate neutrophils *in vitro* directly to produce NETs at low levels (20). Some of these viruses can be detected inside neutrophils, but there is no direct evidence that they establish productive infection in this cell type (20–23). This suggests that pattern recognition receptors (PRRs) expressed on the surface or in endosomes of neutrophils play an essential role in NETosis (Figure 1). For example, neutrophils sense HIV-1 by endosomal PRRs that detect viral nucleic acids, i.e., toll-like receptor (TLR) 7 and TLR8, and subsequently undergo NETosis (17). The fusion protein of respiratory syncytial virus (RSV) induces NETosis through TLR4 (24). NET formation induced by hantaviruses is mediated by signaling through β2 integrins (20). Influenza virus A can also stimulate neutrophils directly to release NETs; however, the molecules involved have not been defined (25). Surprisingly, influenza A virus-induced NETs do not protect against secondary bacterial infection (26). Thus, virus-induced NETs differ structurally and functionally from those generated during bacterial infection. In line with this view, the protein content of NETs depends on the type of NET-inducing stimulus (27).

In the context of viral infection, neutrophils can switch on antiviral effector programs other than NETosis, such as release of antiviral agents or phagocytosis, and can even become apoptotic (18). At the moment, it is unclear how neutrophils decide between these different responses. Possibly, not a single PRR but rather as-yet undefined combinations of neutrophilic PRRs determine the antiviral mode of action of neutrophils. Moreover, only a proportion of cells undergo NETosis, suggesting that only a special neutrophil subtype or maturation stage is susceptible to NETosis induction (4).

Viruses also induce NETosis indirectly without engaging PRRs expressed by neutrophils (Figure 1). The inflammatory milieu created by virus-infected endothelial and epithelial cells contains cytokines and chemokines such as interleukin-8 (IL-8) that trigger NETosis (3, 28, 29). In addition, type I interferon (IFN) is produced in large amounts during viral infections and primes neutrophils for NET formation (30). There is also evidence that platelets play an important role in antiviral defense (31). Platelet activation is frequently observed during viral infections. For example, single-stranded RNA viruses from the family *Picornaviridae* activate platelets through TLR7. This is important for reducing viral titers and increasing the survival of the host (32, 33). Activated platelets form aggregates with neutrophils and in this process stimulate NETosis (34) (Figure 1). On the molecular level, this NET-inducing aggregation has been attributed to surface molecules: CD41 on activated platelets interacts with CD11b, a β2 integrin, on neutrophils. Other infection models have also shown that platelet–neutrophil interactions through β2 integrins induce NET formation (11, 35, 36). Massive activation of the platelet/neutrophil axis and subsequent NET-based clearance mechanisms may represent an emergency strategy of the host in the face of systemically multiplying viruses. This reaction is followed by a drop in platelet counts, which is observed in many viral infections, e.g., viral hemorrhagic fever (VHF) caused by hantaviruses (37, 38). In fact, the degree of platelet loss correlates with the severity of virus-induced disease and determines the clinical outcome (39–41).

## Antiviral Activity of NETs

Although virus induction of NET formation is now well established, it is less clear how NETs contribute to antiviral immunity. In a mouse model of poxvirus infection, induction of NETs with LPS prior to infection strongly reduced the number of virus-infected liver cells and this protective effect was reversed by DNase treatment (34). There are direct mechanisms by which NETs develop antiviral activity (Figure 1). First of all, the web-like chromatin backbone of NETs can bind to and immobilize viral particles, in part by electrostatic attraction, thereby mechanically preventing virus spreading (17). Histones are enriched in positively charged amino acids and can attach to negatively charged viral envelope. For example, the core histones H3 and H4 induce aggregation of seasonal influenza A particles and may inactivate HIV-1 (17, 42). Intriguingly, extracellular histones also reduce HIV-1 transcription (43). Finally, histone H1 binds to noroviruses, the most common cause of viral gastroenteritis and prevents their attachment to intestinal cells (44). Second, attached to the chromatin backbone of NETs are antimicrobial molecules such as myeloperoxidase (MPO), cathelicidins, and α-defensin. They have a proven antiviral activity against both enveloped and non-enveloped viruses and can inactivate viral particles (45).

NETs components also indirectly contribute to antiviral immunity by stimulating antiviral effector mechanisms executed by other immune cells. For example, histones and high mobility group box-1 (HMGB1) proteins act as danger-associated molecular patterns (DAMPs) that trigger release of proinflammatory cytokines and chemokines by other immune cells (46). This process is self-limiting as under high neutrophil densities NETs build aggregates that in turn degrade cytokines and chemokines (47). NETs also activate plasmacytoid dendritic cells (pDCs) through TLRs (48–50). pDCs have a key function in antiviral immunity by releasing high amounts of type I IFN (51). In fact, NETs can be enriched in oxidized mitochondrial DNA which is very efficient in inducing a type I IFN response (52). Finally, NETs could increase antiviral adaptive immunity by reducing the activation threshold of T lymphocytes (53).

## Viral NET Evasion

Viruses are known for their extraordinary capacity to evade immune control mechanisms. There are also viral mechanisms that counteract NET formation (Figure 1). For example, HIV-1 envelope glycoprotein stimulates DCs to produce cellular IL-10 through DC-SIGN (17). IL-10 is an immunosuppressive cytokine that also inhibits TLR-induced ROS production (54). It is quite often produced in the context of viral infections suggesting that more viruses exploit IL-10 as a means of NET evasion (55, 56). In the genome of several large DNA viruses IL-10, homologs have been found including ubiquitous human pathogens such as human cytomegalovirus (HCMV) and Epstein–Barr virus (EBV) (57, 58). As these virus-encoded IL-10 molecules shape the function and cell death of immune cells, they may also modulate NETosis similar to cellular IL-10 (59, 60). Dengue virus (DENV) serotype-2 can arrest NET formation at a ROS-independent late stage by interfering with glucose uptake (61, 62). Finally, latency-associated nuclear antigen 1 encoded by Kaposi’s sarcoma-associated herpesvirus (KSHV) impairs expression of NET-stimulating cellular IL-8 (63).

Some bacteria, such as streptococci, express DNase to degrade NETs (64–66). Herpesviruses also encode proteins that have DNAse activity. These viral molecules process and package the replicated viral genome into the capsid (67). If released from virus-infected cells, they could degrade NETs, thereby remobilizing NET-entrapped virions.

Taken together, virus-induced NETs help to control viral dissemination by several direct and indirect mechanisms, whereas at the same time viral evasion mechanisms target NET formation to minimize the antiviral NET effect and immunopathology.

## Role of NETs in Viral Pathogenesis

As for all effective immune responses against pathogens, NETosis may also result in immunopathology. Unbalanced NET formation is associated with pathological conditions such as respiratory distress, autoimmune disease, and thrombosis (68). NETs are directly cytotoxic to epithelial and endothelial cells (69, 70) as well as hepatocytes (71). They contain several components such as histones that are antimicrobial but at the same time can cause tissue damage and other pathological abnormalities including thrombosis (72). Moreover, NETs can occlude secretory ducts or small airways, thereby driving inflammation (73, 74). Other components of NETs such as HMGB1 may also play a detrimental role in virus-associated disease (75).

There is evidence supporting the concept that local NET deposits contribute to viral immunopathology. NETs have been detected in bronchoalveolar lavage fluid from children with severe RSV infection of the lower respiratory tract (76). Dense plugs occluding the small airways in RSV-infected calves contain NETs (76). Moreover, in a mouse model of influenza pneumonia, NET formation was observed in areas of alveolar-capillary damage in the lung (16). On the other hand, mice deficient in peptidylarginine deiminase 4 (PAD4) were as efficient in controlling influenza virus and showed similar survival as wild-type mice (77). This result suggests that NETs do not play an important role in individual antiviral immunity and virus-induced pathology because PAD4 deiminates histone H3 and H4 and is required for NET formation. The different outcomes of these studies may be due to different virus and mouse strains used. In line with this view, neutrophils from different mouse strains undergo NETosis with different efficiency (78). Furthermore, the influence of NETs on viral dissemination was not addressed in these studies. If virus-induced NET deposits represent an important pathogenic factor treatments that alleviate NET-induced pathological manifestations such as DNase should ease symptoms of virus-associated disease (79). Clinical or radiological improvement after DNase treatment of infants with virus-associated bronchiolitis was observed in some clinical trials (80, 81) but not in others (82). Thus, further studies have to elucidate the precise pathogenic role of virus-induced NET deposits in the lung and explore the efficiency of anti-NET treatment.

NETs start to circulate in detectable amounts in the serum if the NET degradation and clearance machinery of the host is overwhelmed. This systemic NET overflow has severe direct and indirect adverse effects. First, NETs can damage directly endothelial cells lining the interior face of the blood vessels cells (69, 70). Second, NET overflow drives autodestructive processes as components of NETs act as neo self-antigens and induce autoantibodies. In fact, a number of molecules that have been identified as important targets in autoimmune diseases (e.g., dsDNA, histones, MPO, vimentin, and enolase) are actually NET components. Accordingly, NETs have been connected to systemic pathology associated with disease entities such as small vessel-vasculitis, systemic lupus erythematosus (SLE), disseminated intravascular coagulation, rheumatoid arthritis, and preeclampsia (83, 84).

**Figure 2:**
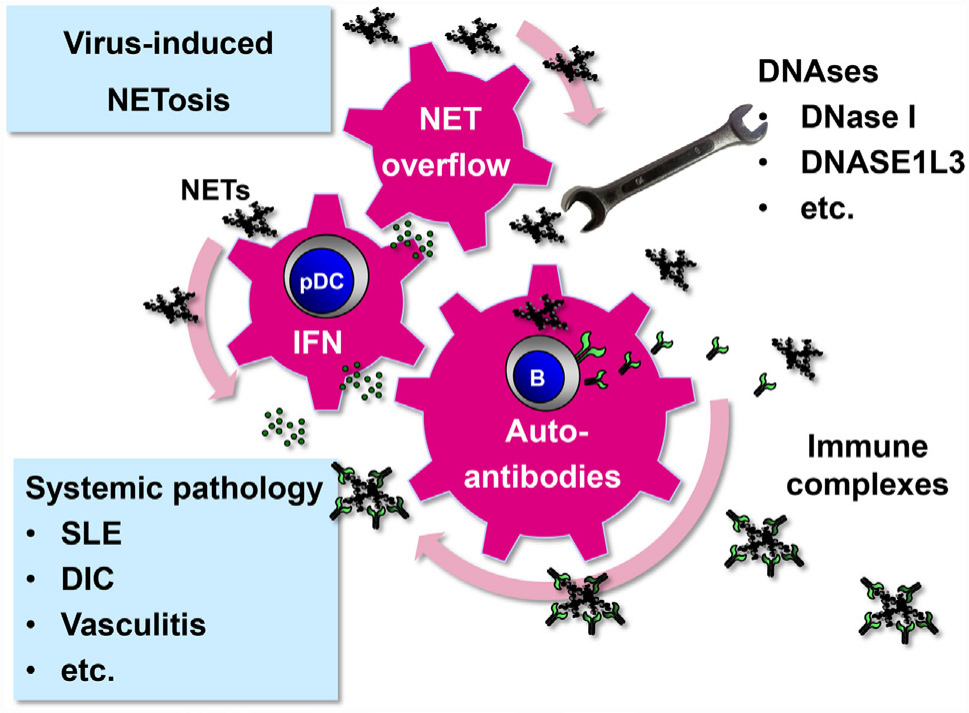
**Systemic pathology driven by virus-induced NET formation**. Virus-induced NETs may start to circulate and become systemic under certain circumstances. First, systemic infection with viruses that have a strong NET-stimulatory capacity, such as hantaviruses, may overwhelm intact NET-degrading function of DNAses (20). Second, persistent viruses with low NET-inducing capacity, such as herpesviruses, may produce systemic NET excess if DNAse activity is compromised. As a result of NET overflow, self-reactive memory B cells are stimulated to release autoantibodies after binding and internalizing NET components through their B cell receptor (91). NETs are enriched in oxidized mitochondrial DNA inducing a strong inflammatory response (52). NETs stimulate pDCs to release type I IFN that adds momentum to the vicious cycle by further activating and expanding autoreactive B cells (48–50). Immune complexes are formed which not only cause systemic pathology as observed in several disease entities such as SLE but also promote the autoimmune process by driving a positive feedback loop.

Systemic NET overflow may result from clearance deficiency or increased NET production. For example, sera from a subpopulation of SLE patients show decreased DNase I activity and NET degradation (85, 86). Another enzyme that could prevent systemic NET overflow is DNASE1L3. It is released by DCs and macrophages and digests microparticle-associated chromatin, thereby preventing SLE (87). In those individuals who are deficient in NET-degrading enzymes even viruses with a relatively weak NETs-stimulatory capacity could drive NET-associated systemic pathology (Figure 2). NET formation represents a plausible link between viruses and systemic autoimmune disease. Supporting this idea, viral infections are associated with transient autoantibody production and are known to mimic SLE, induce SLE onset, or trigger lupus flares (88–90).

Transient systemic NET overflow due to increased NET formation without noticeable deficiency in DNase activity can occur during infection with hantaviruses (20) (Figure 2). Neutrophils play an antiviral role during VHF caused by hantaviruses (92–94). These zoonotic pathogens belong to the family *Bunyaviridae* and infect humans after transmission *via* inhalation of aerosolized urine, saliva, and feces from chronically infected rodents, their natural hosts. In humans, they can induce severe pulmonary and renal dysfunction as well as intravascular coagulation and hemorrhagic shock (95). Hantaviruses replicate in endothelial cells, their main target cells, without causing programed cell death *in vitro*. This suggests that immunopathological mechanisms such as those driven by NETs contribute to Hantavirus-associated pathogenesis (94, 96). In hantavirus-infected patients, high levels of circulating NETs are detected (20). In accordance, increased amounts of cell-free DNA (97) and histones (98) are found in the circulation of hantavirus-infected individuals. The cytotoxic effects of NETs may significantly contribute to hantavirus-associated pathology. In line with this view, histones have been shown to increase thrombin generation and intravascular coagulation (99, 100). They also upregulate the permeability of the endothelial barrier (101). Finally, NETs can induce the formation of autoantibodies that may contribute to the systemic pathology observed during hantavirus-associated disease (20).

Another form of VHF is caused by DENV. DENV is transmitted between humans by *Aedes* mosquitoes and poses a threat to roughly two billion people (102). There is no evidence as yet for a strong direct NET-stimulatory effect of DENV particles *in vitro* (61). Nevertheless, *in vivo* DENV-infected cells could stimulate NETosis indirectly by secreting the viral non-structural protein 1 (NS1). NS1 activates uninfected cells including endothelial cells *via* TLR4 (103, 104). Subsequently, activated endothelial cells could drive neutrophils into NETosis (69, 79). Moreover, NS1 could activate platelets *via* TLR4 which in turn stimulate neutrophils to undergo NETosis (105). Finally, IL-8 is produced by human endothelial cells in response to DENV (29) and is known to drive NETosis (3). In accordance, high levels of IL-8 and elastase, a key component of NETs, are found in DENV patients and correlate with disease severity (106).

These pathological effects explain why NET formation as part of an antiviral defense strategy is a double-edged sword. The host may benefit from NETs deposited precisely in the area of infection, thereby immobilizing or even neutralizing virus and killing virus-infected cells. This benefit may turn into disaster if NET formation is too widespread creating NET deposits in healthy tissue. As a consequence, too many uninfected host cells in the neighborhood of the infected areal may come under “friendly fire” resulting in considerable collateral tissue damage. Local NET-associated pathology may become systemic, if the NET degradation machinery (DNase activity) is impaired, or if the viral NET-stimulatory capacity is too strong. Such an unbalanced NET formation results in NET overflow. Under this condition, autoimmune phenomena are triggered that could result in systemic pathology (Figure 2).

## Concluding Remarks

It is now evident that most pathogens, including viruses, can stimulate neutrophils to undergo NETosis. Although much smaller than bacteria, fungi, or parasites, viral particles do not seem to slip through NETs but rather become immobilized. Whether these viral particles are inactivated as well is a moot point, as long as they are ensnared by NETs, they represent no threat. However, an increasing number of studies indicate that a disproportionate virus-induced NET release can contribute to damage, locally as well as systemically. It will be important to explore the mechanisms that control NET formation in the context of viral infections. On the basis of this knowledge, it could be possible to prevent NET-assisted control of viruses becoming a Pyrrhic victory.

## Author Contributions

Both authors contributed to the conception, writing, and editing of this review.

## Conflict of Interest Statement

The authors declare that the research was conducted in the absence of any commercial or financial relationships that could be construed as a potential conflict of interest.
